# Complete Genome Sequence of Terribacillus aidingensis Strain MP602, a Moderately Halophilic Bacterium Isolated from Cryptomeria fortunei in Tianmu Mountain in China

**DOI:** 10.1128/genomeA.00126-15

**Published:** 2015-03-19

**Authors:** Peng Lu, Mengying Lei, Fenghu Xiao, Liqin Zhang, Yongjun Wang

**Affiliations:** a Key Laboratory of Forest Protection, College of Forestry and Biotechnology, Zhejiang Agriculture and Forestry University, Lin’An, Zhejiang, China; b National Joint Engineering Laboratory of Biopesticide Preparation, Lin’An, Zhejiang, China

## Abstract

Terribacillus aidingensis strain MP602, which was isolated from an ancient tree (Cryptomeria forunei) in Tianmu Mountain in China, has antagonistic activity against several certain phytopathogenic fungi. Here, we report the genome sequence of this strain. This is the first complete genome report of the Terribacillus genus.

## GENOME ANNOUNCEMENT

The genus Terribacillus belongs to the family *Bacillaceae* and was established in 2007 (1). Currently this genus comprises four validly published species: Terribacillus saccharophilus and T. halophilus were isolated from field soil in Japan (1); T. goriensis DSM 18252^T^ was isolated from coastal water of the east coast of Korea (2); and T. aidingensis Y17-61^T^ was isolated from sediments of Aiding Salt Lake in the Xinjiang region of China (3). It has been reported that T. aidingensis was efficient in biological control against gray mold in strawberry (4). Terribacillus aidingensis strain MP602 was isolated from an ∼200-year-old tree (Cryptomeria forunei) in Tianmu Mountain in China. This bacterium displays a moderate tolerance to salts (up to 14%). Interestingly, it showed high antagonistic activity against several plant pathogenetic fungi, such as Botrytis cinerea, Magnaporthe grisea, Rhizoctonia solani, and so on. Therefore, this bacterium has been evaluated as an inoculant for plant disease control. Genome sequencing of T. aidingensis MP602 was conducted to obtain additional insights into physiological characteristics involved in tolerance to salts and plant disease control and for future studies examining the molecular basis of these traits.

The completed genome sequence of T. aidingensis MP602 was developed at the Beijing Genomics Institute (BGI, Beijing, China) using Solexa paired-end sequencing technology (5). Using total genomic DNA, two gene libraries were prepared from sheared DNA fractions (∼500 bp and ∼2 kb) using Illumina paired-end sample preparation kits (Illumina, Inc.) according to the manufacturer’s instructions. The DNA was sequenced using an Illumina Solexa GA IIx instrument. The sequencing run yielded 2,189,000 filtered paired-end reads (500-bp insert; 354 Mb in total) and a total of 3,925,000 reads for the 2-kb inserts (321 Mb in total) that were used for deep sequencing. The total sequence data provided 131-fold coverage of the genome. About 98.2% of the reads were assembled into 20 scaffolds with 251 contigs using the SOAPdenovo alignment tool (http://soap.genomics.org.cn/index.html#intro2). Gaps were filled by Sanger sequencing of PCR products by custom primer walks and long-distance PCR amplification of the regions between each pair of scaffolds and contigs. The open reading frames (ORFs) were predicted using the NCBI Prokaryotic Genomes Automatic Annotation Pipeline (PGAAP) (6), and tRNA and rRNA genes were identified by tRNAscan-SE version 1.3 (7) and RNAmmer version 1.2 (8). The metabolic pathways were examined through the KEGG Automatic Annotation Server (http://www.genome.jp/kegg).

The completed genome of T. aidingensis MP602 contained two replicons, which comprised a circular chromosome (3,488,643 bp) containing 3,587 ORFs and a circular plasmid, which was named pT1 (88,439 bp), carrying 23 ORFs. The GC content of the chromosome is 42.76%, while the content of plasmid pT1 is 37.32%. This genome carried 56 tRNA genes and 24 rRNA operons, which are all located in the chromosome.

### Nucleotide sequence accession numbers.

The complete genome sequence of the T. aidingensis MP602 chromosome is available in GenBank under the accession number CP008876. The GenBank accession number for plasmid pT1 is CP008877.
